# Assessment of Liver Metastases Using CT and MRI Scans in Patients with Pancreatic Ductal Adenocarcinoma: Effects of Observer Experience on Diagnostic Accuracy

**DOI:** 10.3390/cancers12061455

**Published:** 2020-06-03

**Authors:** Masakatsu Tsurusaki, Isao Numoto, Teruyoshi Oda, Miyuki Wakana, Ayako Suzuki, Yukinobu Yagyu, Mitsuru Matsuki, Kazunari Ishii

**Affiliations:** Department of Radiology, Kindai University Faculty of Medicine, Osaka-Sayama 589-8511, Japan; numoto.isao@hotmail.co.jp (I.N.); teru.oda0321@gmail.com (T.O.); miyuki0114ball@yahoo.co.jp (M.W.); i_am_sue_19861211_pinky@yahoo.co.jp (A.S.); y-yagyu@med.kindai.ac.jp (Y.Y.); rad053@poh.osaka-med.ac.jp (M.M.); ishii@med.kindai.ac.jp (K.I.)

**Keywords:** magnetic resonance imaging, multidetector computed tomography, liver, neoplasm metastasis, gadoxetic acid

## Abstract

The aim of this study was to investigate the impact of radiologic experience on the diagnostic accuracy of computed tomography (CT) vs. magnetic resonance imaging (MRI) reporting on the liver metastases of pancreatic ductal adenocarcinoma (LM of PDAC). Intra-individual CT and MRI examinations of 112 patients with clinically proven LM of PDAC were included. Four radiologists with varying years of experience (A > 20, B > 5, C > 1 and D < 1) assessed liver segments affected by LM of PDAC, as well as associated metastases occurring in each patient. Their sensitivity and specificity in evaluating the segments were compared. Cohen’s Kappa (κ) for diagnosed liver segments and Intra-class Correlation Coefficients (ICC) for the number of metastatic lesions in each patient were calculated. The radiologists’ sensitivity and specificity for the CT vs. MRI were, respectively: Reader A—94.4%, 90.3% vs. 96.6%, 94.8%; B—86.7%, 79.7% vs. 83.9%, 82.0%; C—78.0%, 76.7% vs. 83.3%, 78.9% and D—71.8%, 79.2% vs. 64.0%, 69.5%. Reviewers A and B achieved greater agreement in assessing results from the MRI (κ = 0.72, *p* < 0.001; ICC = 0.73, *p* < 0.001) vs. the CT (κ = 0.58, *p* < 0.001; ICC = 0.61, *p* < 0.001), in contrast to readers C and D (MRI: κ = 0.34, *p* < 0.001; ICC = 0.42, *p* < 0.001, and CT: κ = 0.48, *p* < 0.001; ICC = 0.59, *p* < 0.001). Our results indicate that the accurate diagnosis of LM of PDAC depends more on radiologic experience in MRI over CT scans.

## 1. Introduction

International guidelines advise contrast-enhanced computed tomography (CE-CT) for routine diagnosing and staging of pancreatic cancer, whereas magnetic resonance imaging (MRI) is mostly used for the characterization of indeterminate liver lesions [1,2]. The accurate assessment of liver metastases (LM), both colorectal (CRLM) and non-colorectal LM, is crucial in multidisciplinary oncology [1,2,3,4]—especially for patients with pancreatic ductal adenocarcinoma (PDAC). The enhanced detection of LM could reduce the futile resection of tumors and markedly increase life expectancy. 

At present, CE-CT has been widely used as a standard imaging modality to determine the stage of pancreatic cancer. However, its ability to detect LM less than 1 cm in size is reported to be limited and unsatisfactory, given that its rate of accuracy currently stands at just 50% [3,4]. Fortunately, liver-specific magnetic resonance contrast agents like gadoxetate disodium appear to offer great promise because of their ability to provide more precise evaluations of tumor infiltrations [5,6], and are now recommended for the diagnosis and characterization of malignant lesions in non-cirrhotic livers [7]. Moreover, prior studies have demonstrated that multidisciplinary team meetings associate with significant improvements in clinical outcomes, as imaging data is reviewed by all physicians involved in the patient’s care, regardless of their radiologic experience [8,9,10,11]. It is possible that the widespread availability of medical imaging data may eventually lead to independent read-outs of CT and MRI examinations without consultation of the corresponding radiological report. Yet, since marking imagery is not a standardized practice amongst radiologists, but depends more on their reporting preferences and level of experience [12], the comprehensibility of oncologic findings for inexperienced readers remains unknown [2]. 

In this study, we aimed to determine the impact of observer experience in CT and MRI examinations on the diagnostic accuracy of (LM of PDAC).

## 2. Results

### 2.1. Diagnostic Performance

Sensitivity, specificity, positive predictive values (PPV) and negative predictive values (NPV) were calculated on a segmental basis in Table 1. Overall, a trend was found in that the diagnostic performance was proportional to experience for both reporting of CT and MRI examinations in Table 2. In particular, Reviewer A reached the highest sensitivity and specificity for both CT (94.4% and 90.3%, respectively) and MRI (96.6% and 94.8%, respectively), compared with reviewer B (CT: 86.7% and 79.7%, MRI: 83.9 and 82.0%, respectively), C (CT: 78.0% and 76.7%, MRI: 83.3 and 78.9%, respectively), and D (CT: 71.8% and 79.2%, MRI: 64.0 and 69.5%, respectively). Furthermore, Reader A achieved the greatest PPV and NPV for CT (88.6 and 95.7%, respectively) and MRI (96.2 and 95.2%, respectively), compared with Observer B (CT: 73.3 and 90.7%, MRI: 83.2 and 82.7%, respectively), C (CT: 73.1 and 81.2%, MRI: 79.5 and 82.6%, respectively), and D (CT: 77.8 and 73.3%, MRI: 67.3 and 66.6%, respectively). Comprehensive data regarding diagnostic performance are provided in Table 2. The differences between Reviewers A and B, as well as B and D, were significant for CT reporting (*p* = 0.001 for both). Regarding MRI, the following comparisons reached the level of significance, respectively: Readers A and C (*p* = 0.013), A and D (*p* < 0.001), B and D (*p* = 0.001) and C and D (*p* = 0.014). Additional data is summarized in Table 3.

### 2.2. Inter-Observer Agreement

Among all the reviewers, inter-reader agreement for the liver segments that were affected by malignancy was higher for MRI (κ = 0.44, *p* < 0.001) than for CT (κ = 0.43, *p* < 0.001). In particular, the experienced reviewers, A and B, achieved greater agreement for MRI (κ = 0.72, *p* < 0.001) than for CT (κ = 0.58, *p* < 0.001), unlike the less experienced C and D reviewers (MRI: κ = 0.34, *p* < 0.001 and CT: κ = 0.48, *p* < 0.001, respectively). A similar trend was found for the inter-observer agreement regarding the number of LM present in each patient. All reviewers, together, achieved greater inter-observer agreement for MRI (ICC = 0.59, *p* < 0.001) than for CT (ICC = 0.53, *p* < 0.001). The experienced reviewers, A and B, showed higher agreement for MRI (ICC = 0.73, *p* < 0.001) than for CT (ICC = 0.61, *p* < 0.001), as opposed to the less experienced reviewers, C and D (MRI: ICC = 0.41, *p* < 0.001 and CT: ICC = 0.59, *p* < 0.001). Complementary data are shown in Table 4.

## 3. Discussion

This study aimed to investigate the impact of observer experience on diagnostic performance and inter-observer agreement in reporting LM of PDAC using CT and MRI scans because data from these imaging modalities are commonly reviewed by physicians with varying levels of experience in the clinical and radiological practice of oncology [13].

**Figure 1:**
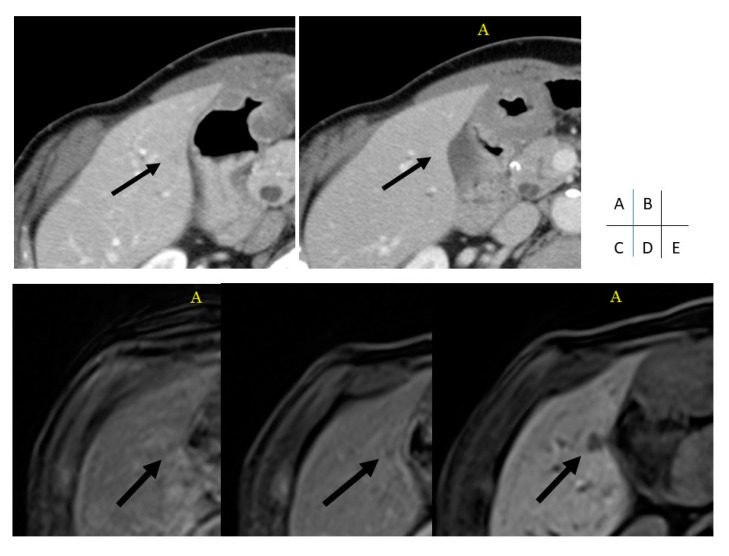
Images of CT and MRI examinations of a 74-year-old female patient with small metastasis of PDAC (A, arterial-phase CT; B, portal-venous phase CT; C, arterial-phase MRI; D, portal-venous phase MRI; E, hepatobiliary phase MRI). A small lesion in segment 5 (arrow) was only detected by the experienced readers, A and B, using MRI (only depicted by hepatobiliary phase MRI), but the lesion was missed by all readers using CT.

We found that diagnostic performance was primarily proportional to reviewer experience, with the most experienced reviewer, A, achieving the highest sensitivity, specificity, PPV and NPV [14,15,16]. Thus, our results indicate that observer experience is essential for exhibiting a high diagnostic accuracy in the aforementioned imaging modalities (Figure 1). More importantly, values indicating diagnostic accuracy differed more distinctly for MRI than for CT, as sensitivity, specificity, PPV and NPV were distributed over a larger range for the MRI analyses [14,15,16]. Therefore, our data suggest that reviewer experience has a greater impact on MRI than on CT reporting. This hypothesis is further supported by the fact that the more experienced radiologists, Reviewers A and B, showed a greater difference in diagnostic performance indices for MRI. This suggests that there is a greater learning curve for MRI interpretation after 5 years of radiologic experience compared with CT reporting. Accordingly, we detected a greater inter-observer agreement among the experienced reviewers for the MRI analyses, both for the amount of LM of PDAC detected per patient and the affected liver segments, as opposed to the less experienced reviewers, who achieved a higher inter-observer agreement using CT images. This may indicate that experienced reviewers are more likely to be consistent in their findings when evaluating MRI scans, unlike less experienced reviewers.

Potential factors that may contribute to the need for more experience in MRI analyses over CT interpretations are the greater number of images that need to be assessed as well as its challenging physical theory. For instance, chemical shift and diffusion-weighted imaging in MRI may be more difficult to assess for less experienced reviewers than CT series that are based on density values and primarily defined by the time of image acquisition relative to the contrast media administration. Contrastingly, standard CT scans for liver imaging in patients with LM of PDAC include a reduced amount of series and often consist solely of a non-contrast and portal-venous phase image acquisition [16]. Thus, our data advocate that the reporting of CT images may be more comprehensible and intuitive for less experienced reviewers. Our results suggest that marking and explaining findings in MRI reports will make them easier to understand for less experienced readers and clinicians, who evaluate imaging data in the absence of an experienced radiologist. Additionally, these results further justify the educational practice in radiological departments wherein residents undergo CT before MRI training. 

Evidence regarding the influence of observer experience in interpreting oncological CT and MRI examinations is scarce, despite the fact that both clinicians and radiologists with varying levels of experience routinely review such studies and thus may affect therapeutic regimens and patient care. Although few investigations have examined the impact of reader experience on the reproducibility of tumor measurements [12,17], a prior study examined the influence of observer expertise on CT and MRI reporting in patients with CRLM [11]. The authors concluded that the MRI analysis of CRLM is more affected by observer experience than CT interpretation [11]. Our results are consistent with this hypothesis and likewise indicate that reviewer experience is a crucial determinant in diagnostic accuracy. More importantly, they also support the assumption that this effect is more distinct within MRI over CT reporting. Therefore, we conclude that experience may have a greater impact on diagnostic accuracy for the MRI reporting of LM of varying primary carcinomas compared with CT analyses. In addition to this prior investigation, we found consistent results using CT and MRI examinations on the same individuals, which included a diffusion-weighted MRI series.

This study has limitations that should be mentioned. First, we only included four radiologists with varying levels of experience. Because the influence of experience on diagnostic accuracy is challenging to investigate, results may be more representative following a larger population of reviewers in future studies. Additionally, subtle changes of LM of PDAC that were not indicated by criteria in the image analyses may limit the comparability of the intra-individual CT and MRI examinations.

## 4. Materials and Methods

### 4.1. Patients

This retrospective study was approved by our local institutional review board (Ethical Committee of Kindai University No. 23-101), and written informed consent was obtained from each patient before undergoing a CT or MRI scan. This study used data from clinical records and images collected from Kindai University Hospital, a high-volume regional referral center. Between January 2009 and December 2017, patients with histologically confirmed pancreatic cancer who underwent a CE-CT and gadoxetic acid-enhanced MRI were enrolled in the study. CT and MRI examinations of 164 patients with LM of PDAC were retrospectively included. From the initial study group, 52 patients were excluded because their time intervals between the CT and MRI examination were greater than one month, so 112 individuals in total participated (62 males, mean age ± standard deviation: 62 ± 12.4 y). There were 172 of LM of PDAC and 157 liver segments that were affected by LM of PDAC. 

### 4.2. CT Imaging Protocol

Intravenous CE-CT imaging was performed using a 64-channel multidetector row scanner (Light Speed VCT Vision, GE Healthcare, Waukesha, WI, USA) with a tube voltage of 100-170 kV, an automatic dose modulation, a pitch of 2.0 and a slice thickness of 1 mm. Axial, coronal and sagittal slices were reconstructed with a section thickness of 5.0 mm and an increment of 3 mm. After the unenhanced images were acquired, 510 mg/kg of iodinated contrast material (Optiray 320, Guerbet Japan, Tokyo, Japan) was administered intravenously into the antecubital vein at a rate of 3–4 mL/s. Scanning was performed at the beginning of the pancreatic parenchymal phase (after 40 s) and the subsequent liver phase was obtained 70 s after the intravenous administration of the contrast material.

### 4.3. MR Imaging Protocol

Enhanced magnetic resonance imaging (EOB-MRI) was performed with two superconducting magnet systems on a 3.0-T scanner (Magnetom Trio, Siemens Medical Systems, Erlangen, Germany; *n* = 48; Achieva TX, Philips Healthcare, Best, The Netherlands; *n* = 64) using a 32-channel phased-array body coil for all patients. The Magnetom Trio scanner was actively shielded with a 45 mT/m gradient field strength and slew rate of 200 T/m/s. The Achieva TX scanner was actively shielded with a 50 mT/m gradient field strength and slew rate of 220 T/m/s. FOr both scanners, after breath-hold double-echo T1-weighted gradient recalled echo (GRE) images (in-phase and opposed-phase images) and navigator-triggered fat-suppressed T2-weighted turbo spin-echo (TSE) images were obtained, dynamic fat-suppressed T1-weighted images were obtained with a three-dimensional (3D) GRE sequence before (pre-contrast), 14–30 s after (arterial phase by means of a bolus-triggered technique), 70 s after and 3 min after the intravenous administration of gadoxetic acid (EOB Primovist; Bayer Yakuhin, Osaka, Japan), which was injected as a bolus (2.0 mL/s) at a dose of 0.025 mmol/kg of body weight, followed by a 20 mL saline flush. Hepatocyte-phase images were obtained 20 min after the gadoxetic acid injection.

The Siemens scanner acquired breath-hold T1-weighted GRE images (a repetition time/echo time (TR/TE) of 120/2.46 ms, a flip angle of 66°, a matrix size of 320 × 180, a bandwidth of 434.3 Hz/pixel, one signal acquired, a section thickness of 7 mm, an intersectional gap of 1.4 mm and an acquisition time of 28 s), navigator-triggered fat-suppressed T2-weighted TSE images using Prospective Acquisition Correction (PACE) (a TR/TE of 3865–5534/71 ms, a flip angle of 120°, a matrix size of 384 × 202, a bandwidth of 506.4 Hz/pixel, one signal acquired, a section thickness of 7 mm, an intersectional gap of 1.4 mm and an acquisition time of approximately 90 s). For the EOB-MRI, fat-suppressed T1-weighted GRE images were acquired with a 3D-Volumetric Interpolated Breath-hold Examination (VIBE) sequence (a TR/TE of 3.68/1.22 ms, a flip angle of 10°, a matrix size of 256 × 192, one signal acquired, a section thickness of 3 mm, an intersectional gap of 0.6 mm and an acquisition time of 21 s. 

The Philips scanner included a respiration-triggered T1-weighted turbo field-echo in-phase sequence (a TR/TE of 10/2.3 ms, a flip angle of 15°, a matrix size of 288 × 230, a bandwidth of 434.3 Hz/pixel, one signal acquired, a section thickness of 7 mm, an intersectional gap of 1.2 mm and an acquisition time of 26 s) and a respiration-triggered fat-suppressed T2-weighted sequence with a reduction factor of 2 or 4 (a TR/TE of 1342/80 ms, a flip angle of 90°, a matrix size of 320 × 256, a bandwidth of 506.4 Hz/pixel, one signal acquired, a section thickness of 7 mm, an intersectional gap of 1.2 mm and an acquisition time of approximately 100 s). For the EOB-MRI, a fat-suppressed T1-weighted 3D turbo field-echo sequence was performed (T1 high-resolution isotropic volume examination [THRIVE], Philips Healthcare) (a TR/TE of 3.4/1.8 ms, a flip angle of 10°, a matrix size of 336 × 206, a bandwidth of 995.7 Hz/pixel) with a 3 mm section thickness and a field-of-view of 32–38 cm.

### 4.4. Image Analysis

All CT and MRI series were reviewed by four reviewers with varying levels of experience in oncologic radiology (Reviewer A > 20 y, B > 15 y, C > 1 y, D < 1 y) according to Albrecht ‘s methodology [11]. They were aware that this study focused on the detection of LM, though were not told that LM of PDAC was present in each patient and were blinded to patients’ ages, primary cancers, clinical course and previous treatments. Images were assessed in a randomized order, with a mandatory time interval between the intra-individual read-outs of CT and MRI datasets of one week, so as to reduce potential recall biases. Preset window settings could be freely adjusted. Reviewers reported the amount of metastases they detected per patient, as well as the liver segments that were affected by LM of PDAC, according to the Couinaud classification of hepatic anatomy. Segment 4 was evaluated as a cranial (4a) and caudal portion (4b). Thus, each liver segment was either rated as affected (positive) or not affected (negative) regarding malignant infiltration by LM of PDAC. 

The criteria for the radiological diagnosis of LM on the CE-CT were that they had to be ill-defined, heterogeneous nodules with a higher attenuation than bile and having some degree of enhancement. The criteria for the radiological diagnosis of LM on the EOB-MRI were that they had to be focal, discrete nodular lesions that showed a high signal intensity relative to the liver parenchyma on T2-weighted fast spin-echo (FSE) images (with a lower signal intensity than the gallbladder or cerebrospinal fluid), a low signal intensity relative to the liver parenchyma on T1-weighted GRE images obtained at 70 s and 3 min after the gadoxetic acid injection, and were more conspicuous on the hepatocyte-phase images. The diagnosis of LM was more definite when perilesional enhancement was detected on the T1-weighted GRE images obtained 30 s after the gadoxetic acid injection (Figure 1).

### 4.5. Standard of Reference

In patients who underwent endoscopic ultrasonography fine-needle aspiration (EUS-FNA) for liver tumors, the diagnosis of LM was made based on a combination of the histopathological findings of EUS-FNA samples and follow-up imaging examinations. In cases without histopathological diagnosis, the final diagnosis was confirmed by combining all available imaging examinations performed 2 to 3 m after the initial CT and MRI. If possible, observation with these imaging examinations of the lesions was followed up every 2 mos. In cases lacking a histopathological diagnosis, the final diagnosis was made when a significant change in the tumor’s size was confirmed using these imaging examinations. Most of the tumors (159 tumors) were increased in the interval. A few tumors were confirmed to have shrunk by chemotherapy and were not abscesses by combining all available imaging examinations. In this study, LM were considered to pre-exist at the time of the initial examination, despite no detection by any imaging modalities (metastatic lesions were identified by follow-up imaging within 3 m of the initial examination).

### 4.6. Statistical Analysis

Statistical evaluation was performed using dedicated software (MedCalc, v. 12.7.2 and BiAS, v. 10.11); *p*-value < 0.05 was considered to indicate a statistically significant difference. Segment-based sensitivity, specificity, positive predictive values (PPVs) and negative predictive values (NPVs) were compared using the McNemar test. Confidence intervals (CI) of 95% were calculated. For the qualitative lesion- and segment-based analysis, Cohen’s kappa (κ) statistics were performed. The values were interpreted as follows: κ < 0.40, poor agreement; κ = 0.40–0.75, fair to good agreement; κ > 0.75, excellent agreement [18] The quantitative analysis for the number of metastases detected per patient was conducted using Intraclass Correlation Coefficients (ICC). ICC values were classified as follows: ICC < 0.40, poor agreement; ICC = 0.40–0.60, moderate agreement; ICC = 0.60–0.80, substantial agreement; ICC > 0.80, good agreement [19].

## 5. Conclusions 

Our results indicate that the accurate diagnosis of LM of PDAC depends more on radiologic experience in MRI than in CT scans. Although observer experience is crucial for a high diagnostic accuracy regarding both CT and MRI analysis in patients with LM of PDAC, this effect seems to be more pronounced in MRI reporting.

## Figures and Tables

**Table 1:** Mean and standard deviation (SD) of segment-based sensitivity, specificity, positive predictive values (PPV) and negative predictive values (NPV) for CT and MRI assessments

	CT		MRI	
	Mean	SD	Mean	SD
**Sensitivity**	82.76	8.55	82.15	11.65
**Specificity**	81.51	5.21	81.34	9.06
**PPV**	78.24	6.30	81.54	10.41
**NPV**	85.27	8.62	81.84	10.15

**Table 2:** CT and MRI sensitivity, specificity, positive predictive value (PPV) and negative predictive value (NPV) on a segmental base achieved by reviewers A (> 20 y of experience), B (> 10 y), C (> 1 y), and D (< 1 y). Diagnostic performance was mainly proportional to radiological experience.

CT									MRI								
	Reader A	95%-CI	Reader B	95%-CI	Reader C	95%-CI	Reader D	95%-CI	Reader A	95%-CI	Reader B	95%-CI	Readr C	95%-CI	Reader D	95%-CI
**Sensitivity (%)**	94.44	91.7–97.78	86.77	81.9–92.28	78.04	72.9–84.34	71.80	66.5–78.58	96.63	92.2–97.79	83.99	77.5–87.55	83.39	76.7–87.10	64.02%	56.2–69.42
**Specificity (%)**	90.36	86.0–93.73	79.77	74.3–84.50	76.71	70.9–81.81	79.25	73.1–84.50	94.85	91.1–97.31	82.05	76.5–86.75	78.93	73.2–83.89	69.53%	63.1–75.37
**PPV (%)**	88.65	85.0–93.26	73.37	67.8–80.24	73.10	67.8–79.71	77.84	72.6–84.19	96.25	91.83–97.52	83.29	76.81–86.92	79.58	72.8–83.61	67.04%	59.1–72.42
**NPV (%)**	95.74	92.3–97.94	90.71	86.1594.16	81.28	75.69–86.05	73.36	67.1–78.97	95.26	91.67–97.61	82.76	77.2–87.39	82.68	77.1–87.33	66.67%	60.36–72.56

CI, confidence interval; PPV, positive predictive value; NPV, negative predictive value.

**Table 3:** Statistical comparisons of diagnostic performance were performed using the McNemar test.

Reviewers	CT (*p*-Value)	MRI (*p*-Value)
**A + B**	***0.0015***	0.0763
**C + D**	0.0768	***0.0138***

Significant values (*p* < 0.05) are given in bold italics.

**Table 4:** Inter-observer agreement regarding liver segments that were affected by the liver metastases of pancreatic ductal adenocarcinoma (LM of PDAC) (κ) and the number of lesions reported per patient (ICC) for CT and MRI analyses. The experienced reviewers showed a significantly higher agreement for MRI than for CT reporting.

Data	Κ		ICC	
Reviewers	CT	MRI	CT	MRI
A + B + C + D	0.43	0.44	0.53	0.59
A + B	0.58	0.72	0.61	0.73
C + D	0.48	0.34	0.59	0.42
